# Supplementary material for: A Likelihood Approach for Real-Time Calibration of Stochastic Compartmental Epidemic Models
Source: PLoS Comput Biol. 2017 Jan 17;13(1):e1005257. doi: 10.1371/journal.pcbi.1005257 (PMC5240920; doi:10.1371/journal.pcbi.1005257)
Supplement: S1 File — (TAR.GZ) [file pcbi.1005257.s014.tar.gz › HSPH_Online-SI-Revision/EnKF07/n1-extreme/n1-extreme_EnKF_table4.pdf]

1	R0	{2.95019, 14.2854}	{2.5345, 6.32883}	{{1.72828, 2.80288}, {3., 11.2839}}	{15.8035, 60.4496}	inf	101.	46.508	{7.42466, 50.4932}	93.4914	0.776173	newly	40.	40.93	{4., 56.}	104.975	0.51845	Reff	0.31272	0.397268	{0.113911, 0.598116}	56.1911	ar	924.	1711.16	{1580., 1833.}	85.1905
2	R0	{2.67565, 16.946}	{2.47131, 9.32348}	{{0.940495, 3.97357}, {3., 16.609}}	{24.6083, 46.0427}	inf	81.	69.3198	{12.6373, 125.823}	80.7956	0.451248	newly	24.	53.14	{8., 115.}	163.167	0.363145	Reff	0.294322	0.479296	{0.127429, 0.689899}	97.0349	ar	926.	1727.49	{1550., 2096.}	86.554
3	R0	{2.40066, 16.6543}	{2.77114, 7.3714}	{{2.37652, 3.66713}, {3., 9.26118}}	{16.3626, 56.2711}	inf	164.	45.6591	{9.48131, 55.6326}	89.4465	0.871746	newly	51.	40.6	{6., 50.}	96.3529	0.550182	Reff	0.40091	0.528137	{0.189532, 0.652109}	56.2774	ar	916.	1753.7	{1594., 2164.}	91.452
4	R0	{1.9294, 15.4361}	{2.55293, 6.0641}	{{1.89318, 3.22549}, {3., 9.66419}}	{33.4852, 60.7148}	inf	105.	42.9549	{4.3229, 34.3528}	91.055	0.826823	newly	40.	48.33	{4., 83.}	130.025	0.585996	Reff	0.677219	0.463028	{0.140158, 0.657771}	54.0015	ar	796.	1678.13	{1551., 1968.}	110.82
5	R0	{2.92859, 19.5393}	{2.70688, 2.78198}	{{1.78546, 1.29723}, {3., 4.48531}}	{11.033, 85.7621}	inf	114.	45.3478	{3.62111, 46.3582}	85.1964	0.778229	newly	40.	83.14	{7., 140.}	165.1	0.394938	Reff	0.351431	0.53086	{0.277052, 0.852585}	63.7788	ar	927.	1589.52	{1446., 1911.}	71.4693
6	R0	{1.79622, 12.3148}	{2.42824, 8.67912}	{{0.0889966, 5.99772}, {3., 13.3312}}	{65.9728, 32.9494}	inf	56.	78.1431	{6.93339, 64.9977}	127.336	0.461706	newly	31.	51.37	{4., 45.}	139.194	0.432499	Reff	0.661009	0.378571	{0.0112722, 0.490695}	61.2417	ar	734.	1753.7	{1568., 2123.}	138.924
7	R0	{2.45855, 17.1776}	{2.34324, 7.58724}	{{0.818035, 4.22051}, {3., 10.6903}}	{31.042, 55.8306}	inf	121.	34.9124	{2.11842, 70.6362}	84.4396	0.945563	newly	52.	38.58	{2., 71.}	109.077	0.760241	Reff	0.486793	0.47411	{0.130557, 0.80024}	48.515	ar	871.	1743.46	{1597., 2249.}	100.168
8	R0	{2.20009, 15.519}	{2.43126, 5.59148}	{{1.34094, 0.680832}, {3., 10.8311}}	{35.5479, 63.9701}	inf	65.	96.041	{5.02463, 353.135}	134.341	0.491763	newly	25.	115.51	{6., 384.}	401.24	0.466182	Reff	0.448818	0.479957	{0.122617, 0.948697}	64.5543	ar	839.	1805.76	{1572., 2588.}	115.228
9	R0	{2.06493, 9.6414}	{2.5626, 5.48569}	{{1.75976, 3.4144}, {3., 5.93394}}	{33.6039, 46.4983}	inf	54.	33.6317	{4.06256, 53.9586}	93.5616	0.641078	newly	29.	47.25	{4., 48.}	155.276	0.496571	Reff	0.580245	0.519528	{0.117362, 0.891233}	48.103	ar	777.	1720.04	{1569., 2119.}	121.369
10	R0	{2.60775, 19.6936}	{2.55516, 3.94539}	{{1.37465, 1.79371}, {3., 5.40811}}	{21.8474, 79.9661}	inf	139.	23.092	{3.05339, 27.1836}	94.3787	1.15583	newly	45.	31.61	{5., 38.}	101.4	0.67469	Reff	0.47461	0.495406	{0.150453, 0.908522}	47.2958	ar	889.	1555.57	{1467., 1677.}	74.9798
11	R0	{1.99168, 8.13555}	{2.27594, 7.94735}	{{0.830172, 3.94412}, {3., 12.6662}}	{49.2551, 35.3438}	inf	45.	44.6097	{8.91185, 70.6835}	87.343	0.406879	newly	31.	37.51	{6., 57.}	108.161	0.442455	Reff	0.579579	0.390958	{0.07441, 0.747504}	46.8803	ar	772.	1717.89	{1588., 1932.}	122.525
12	R0	{1.79304, 9.18941}	{2.57617, 6.12108}	{{1.75598, 3.26354}, {3., 9.12055}}	{44.8064, 39.142}	inf	52.	40.0782	{6.76051, 37.7354}	91.9604	0.514285	newly	36.	29.93	{5., 42.}	83.6389	0.452003	Reff	0.627564	0.402107	{0.13877, 0.600059}	46.9732	ar	725.	1691.81	{1587., 1722.}	133.353
13	R0	{1.84016, 18.3431}	{2.40914, 4.49317}	{{1.4416, 0.741589}, {3., 7.1563}}	{46.1182, 75.5049}	inf	88.	50.0213	{5.1384, 76.0153}	94.2141	0.724083	newly	32.	86.48	{5., 190.}	240.5	0.501222	Reff	0.875916	0.585326	{0.0859388, 1.14496}	56.1487	ar	762.	1757.23	{1537., 2101.}	130.608
14	R0	{1.94221, 9.85971}	{2.61594, 10.445}	{{1.91445, 3.55036}, {3., 16.4681}}	{35.4534, 42.7408}	inf	31.	69.8368	{13.1561, 120.969}	149.058	0.372593	newly	20.	49.98	{5., 105.}	182.2	0.41961	Reff	0.42146	0.611774	{0.121755, 1.76136}	86.6904	ar	805.	1808.81	{1576., 2368.}	124.697
15	R0	{1.78578, 12.0541}	{2.40149, 3.48403}	{{0.0878154, 1.0573}, {3., 7.04485}}	{72.1874, 74.1008}	inf	61.	21.2045	{1.9493, 51.4802}	80.5877	0.839656	newly	32.	32.39	{2., 84.}	108.031	0.625888	Reff	0.558949	0.429739	{0.0198453, 0.910116}	58.8353	ar	732.	1578.6	{1482., 1751.}	115.656
16	R0	{1.94463, 15.6503}	{2.28043, 4.79439}	{{0.852614, 0.141936}, {3., 8.07752}}	{51.1191, 69.9077}	inf	82.	60.0158	{4.52399, 98.9407}	94.7854	0.631465	newly	29.	78.9	{4., 160.}	232.69	0.472638	Reff	0.663119	0.418802	{0.0481641, 0.836747}	52.957	ar	757.	1734.59	{1575., 2122.}	129.14
17	R0	{2.08901, 16.4314}	{2.42694, 5.82043}	{{1.11753, 2.47563}, {3., 8.18714}}	{41.149, 64.7492}	inf	116.	61.207	{10.2521, 89.2461}	85.1341	0.639219	newly	42.	77.99	{8., 203.}	159.548	0.422158	Reff	0.676839	0.488941	{0.0901212, 0.748342}	50.4408	ar	826.	1713.84	{1536., 2259.}	107.487
18	R0	{2.76419, 18.56}	{2.29198, 7.09867}	{{0.792651, 4.87618}, {3., 10.3558}}	{26.6324, 61.7529}	inf	153.	36.5485	{8.69479, 58.2301}	83.5119	0.85664	newly	51.	35.72	{6., 53.}	93.2941	0.580095	Reff	0.351052	0.439231	{0.0527612, 0.747078}	75.5372	ar	920.	1691.91	{1566., 1864.}	83.9033
19	R0	{2.3821, 17.1399}	{2.63319, 7.29884}	{{1.77344, 6.60162}, {3., 10.3308}}	{24.7006, 57.4161}	inf	162.	99.27	{12.0298, 114.62}	89.2308	0.639363	newly	73.	83.64	{9., 107.}	106.74	0.476115	Reff	0.443071	0.470948	{0.105613, 0.708435}	47.5543	ar	883.	1852.58	{1616., 2422.}	109.805
20	R0	{2.78314, 19.6127}	{2.37235, 6.67834}	{{1.29245, 3.36191}, {3., 7.97207}}	{23.6427, 65.9489}	inf	71.	58.3527	{9.32724, 92.135}	81.7686	0.460722	newly	24.	73.71	{7., 216.}	250.375	0.396713	Reff	0.222651	0.479894	{0.113686, 0.752852}	141.115	ar	943.	1706.85	{1520., 2246.}	81.0021
21	R0	{1.85124, 14.6794}	{2.4953, 6.27996}	{{1.19311, 3.65707}, {3., 8.97254}}	{51.0258, 57.2192}	inf	108.	46.0484	{4.811, 44.5166}	90.1258	0.758412	newly	46.	46.74	{4., 50.}	114.87	0.561946	Reff	0.794182	0.392165	{0.101968, 0.734166}	65.961	ar	730.	1694.94	{1552., 1901.}	132.184
22	R0	{2.1028, 14.9852}	{2.59433, 5.61088}	{{1.95383, 2.11045}, {3., 9.19196}}	{28.4406, 62.5572}	inf	84.	38.1223	{5.82573, 60.029}	74.7209	0.624485	newly	32.	65.59	{5., 150.}	176.531	0.456302	Reff	0.471027	0.603394	{0.0734416, 1.82433}	79.5031	ar	840.	1762.82	{1565., 2385.}	109.86
23	R0	{1.86601, 10.2157}	{2.33148, 6.93235}	{{0.848522, 2.77046}, {3., 13.307}}	{54.2404, 42.4838}	inf	60.	37.7234	{4.88059, 65.8922}	87.125	0.597125	newly	37.	39.07	{3., 54.}	107.919	0.535762	Reff	0.649371	0.532304	{0.0550552, 0.954625}	56.5053	ar	747.	1732.88	{1566., 2068.}	131.979
24	R0	{2.34032, 10.0992}	{2.52127, 6.41741}	{{1.75047, 3.30787}, {3., 12.9453}}	{24.9273, 49.3828}	inf	58.	45.2452	{4.77021, 32.711}	105.558	0.62534	newly	35.	33.29	{3., 40.}	96.3714	0.504318	Reff	0.355729	0.423795	{0.15335, 0.643482}	54.0723	ar	887.	1708.14	{1592., 1767.}	92.575
25	R0	{1.91548, 15.3401}	{2.60134, 4.66993}	{{1.90422, 2.55541}, {3., 8.05205}}	{36.2458, 69.5574}	inf	102.	62.9387	{7.70848, 99.0117}	79.8115	0.547011	newly	45.	91.08	{8., 192.}	159.511	0.375147	Reff	0.739375	0.529898	{0.0658142, 1.35357}	69.9717	ar	765.	1772.92	{1577., 2349.}	131.754
26	R0	{2.75283, 10.6721}	{2.1611, 6.04199}	{{0.671794, 1.04965}, {3., 12.5755}}	{31.5646, 50.2786}	inf	40.	69.38	{7.72578, 122.171}	134.918	0.362092	newly	22.	61.86	{5., 140.}	220.909	0.406389	Reff	0.286294	0.398063	{0.0753153, 0.693797}	85.3029	ar	926.	1738.95	{1573., 2088.}	87.7916
27	R0	{2.08367, 19.7849}	{2.68349, 5.78115}	{{2.33644, 1.1617}, {3., 11.7173}}	{34.4157, 70.78}	inf	132.	58.7037	{4.42167, 106.433}	82.9998	0.75667	newly	42.	72.88	{3., 120.}	147.619	0.486067	Reff	0.614683	0.513934	{0.190903, 0.92657}	56.6854	ar	833.	1759.19	{1556., 2418.}	111.187
28	R0	{2.64334, 17.1765}	{2.71626, 4.73022}	{{2.37742, 1.1193}, {3., 8.68999}}	{13.6051, 73.8331}	inf	110.	28.346	{1.92386, 25.0529}	84.5474	0.905181	newly	34.	45.98	{3., 44.}	121.353	0.482645	Reff	0.311914	0.539769	{0.152755, 0.715561}	91.9364	ar	934.	1672.07	{1545., 1764.}	79.0225
29	R0	{2.87008, 13.3872}	{2.72658, 4.73993}	{{2.40056, 1.68409}, {3., 5.03954}}	{10.6129, 68.2698}	inf	80.	19.2991	{3.38189, 28.7456}	82.2691	0.852943	newly	36.	28.35	{3., 33.}	92.3611	0.561008	Reff	0.226736	0.500332	{0.200524, 0.874796}	128.023	ar	941.	1608.22	{1510., 1798.}	70.9054
30	R0	{2.06105, 19.6275}	{2.68409, 6.60128}	{{1.89484, 2.40254}, {3., 15.5887}}	{37.1948, 66.3672}	inf	42.	52.7509	{5.5334, 41.8542}	111.636	0.422948	newly	18.	59.89	{3., 61.}	280.056	0.384517	Reff	0.595643	0.441061	{0.0829594, 0.599818}	60.689	ar	747.	1646.24	{1477., 2024.}	120.38
31	R0	{2.30764, 13.435}	{2.15717, 5.18538}	{{0.742215, 3.12607}, {3., 5.92806}}	{40.3107, 62.3438}	inf	82.	47.5301	{7.9319, 91.9342}	86.3078	0.608412	newly	42.	62.85	{6., 125.}	139.881	0.455078	Reff	0.376145	0.453365	{0.0915435, 0.725012}	64.6557	ar	877.	1772.48	{1625., 2146.}	102.107
32	R0	{2.30565, 11.9237}	{2.23546, 7.48182}	{{0.757853, 3.17551}, {3., 12.9226}}	{38.5433, 42.2296}	inf	65.	53.2521	{8.83722, 45.8752}	95.1625	0.496398	newly	34.	44.33	{4., 39.}	115.265	0.401928	Reff	0.378127	0.382238	{0.0660547, 0.626217}	58.8924	ar	853.	1772.91	{1597., 2264.}	107.844
33	R0	{2.24031, 18.582}	{2.73041, 5.94787}	{{2.39654, 0.444955}, {3., 9.65806}}	{26.3103, 67.9912}	inf	156.	45.7785	{5.41729, 55.159}	85.1633	0.830228	newly	47.	81.9	{5., 87.}	163.66	0.491627	Reff	0.578	0.561013	{0.242713, 0.824213}	53.9506	ar	864.	1664.01	{1511., 1661.}	92.5938
34	R0	{2.33306, 15.9759}	{2.756, 7.63344}	{{2.39044, 6.56559}, {3., 10.2331}}	{20.6777, 52.5041}	inf	110.	76.5241	{9.11443, 100.844}	88.2875	0.584454	newly	38.	68.3	{6., 103.}	145.842	0.418934	Reff	0.468945	0.604846	{0.213078, 0.879565}	52.7981	ar	854.	1792.01	{1577., 2068.}	109.837
35	R0	{2.48357, 18.3573}	{2.30163, 6.92653}	{{0.806507, 3.43265}, {3., 8.02788}}	{32.2496, 62.2683}	inf	120.	77.9423	{7.98508, 191.374}	99.4874	0.721546	newly	42.	69.27	{5., 204.}	156.643	0.518941	Reff	0.588606	0.498656	{0.0814747, 0.833555}	51.8913	ar	835.	1798.79	{1592., 2448.}	115.424
36	R0	{2.59548, 19.9046}	{2.46871, 9.32468}	{{1.16502, 6.79023}, {3., 14.8326}}	{26.1159, 53.1531}	inf	123.	74.7226	{6.53037, 96.1927}	85.7254	0.636855	newly	46.	53.87	{4., 70.}	106.674	0.515296	Reff	0.490546	0.550518	{0.127612, 1.11507}	57.9882	ar	863.	1768.58	{1546., 2322.}	104.934
37	R0	{2.29641, 16.9868}	{2.76348, 6.62338}	{{2.23904, 1.95103}, {3., 18.13}}	{23.1678, 63.6166}	inf	111.	23.8841	{4.94568, 30.7847}	86.398	0.939771	newly	31.	25.51	{3., 52.}	77.3871	0.488385	Reff	0.420243	0.653656	{0.0569339, 2.2368}	101.406	ar	877.	1644.69	{1452., 2292.}	87.5359
38	R0	{2.65743, 15.4569}	{2.50577, 8.1282}	{{1.11175, 4.07855}, {3., 12.7557}}	{24.0124, 47.4167}	inf	85.	82.3221	{11.4962, 102.492}	95.4351	0.464794	newly	40.	64.21	{8., 160.}	128.325	0.390094	Reff	0.385327	0.477222	{0.0923627, 0.722523}	71.2936	ar	897.	1767.7	{1572., 2406.}	97.068
39	R0	{2.67953, 14.5132}	{2.25698, 5.85669}	{{0.731328, 0.515488}, {3., 6.55544}}	{29.6432, 62.3226}	inf	119.	55.6319	{6.72025, 127.427}	74.0937	0.644794	newly	46.	63.02	{5., 152.}	116.261	0.456378	Reff	0.404609	0.426039	{0.0844736, 0.606073}	56.8376	ar	894.	1760.02	{1603., 2143.}	96.8702
40	R0	{1.94594, 11.928}	{2.59581, 4.65476}	{{1.96846, 2.07932}, {3., 8.19336}}	{33.3989, 60.9762}	inf	60.	41.5009	{2.27838, 82.5776}	82.819	0.638042	newly	31.	77.56	{2., 160.}	224.903	0.587354	Reff	0.558485	0.617839	{0.171342, 1.95115}	73.6748	ar	796.	1768.15	{1572., 2378.}	122.129
41	R0	{1.66297, 10.0047}	{2.37221, 3.54545}	{{0.247624, 0.748379}, {3., 2.90924}}	{81.167, 79.1087}	inf	53.	21.752	{2.06301, 53.84}	80.1394	0.764644	newly	33.	31.65	{3., 80.}	102.212	0.589621	Reff	0.640243	0.386996	{0.0338337, 0.836258}	69.9233	ar	716.	1618.55	{1510., 1943.}	126.054
42	R0	{2.91568, 16.4453}	{2.52084, 6.56951}	{{1.77098, 2.16034}, {3., 9.56949}}	{16.7511, 60.0524}	inf	138.	52.4865	{5.64497, 81.1253}	87.8472	0.813822	newly	55.	68.75	{4., 131.}	142.6	0.627796	Reff	0.297399	0.521686	{0.0810121, 0.88589}	103.627	ar	919.	1752.15	{1557., 2067.}	90.6583
43	R0	{2.55898, 15.8889}	{2.38017, 5.0264}	{{0.859482, 0.216994}, {3., 6.8674}}	{27.4855, 68.684}	inf	147.	39.1849	{5.32199, 71.408}	79.327	0.851787	newly	62.	52.42	{5., 91.}	94.9032	0.625484	Reff	0.455498	0.45463	{0.120225, 0.871626}	53.3186	ar	904.	1710.77	{1585., 1994.}	89.2445
44	R0	{2.28649, 15.9017}	{2.55136, 6.10434}	{{1.88046, 3.23365}, {3., 7.54775}}	{25.0282, 62.4603}	inf	92.	52.9483	{7.11648, 83.8123}	84.7348	0.668058	newly	43.	50.04	{4., 121.}	101.535	0.532221	Reff	0.400136	0.50168	{0.0984021, 1.08835}	72.8142	ar	880.	1742.66	{1565., 2359.}	98.0295
45	R0	{2.65121, 18.6339}	{2.55149, 6.19301}	{{1.78258, 2.2523}, {3., 9.35997}}	{18.7935, 66.7648}	inf	160.	34.9899	{6.91177, 49.9036}	84.127	0.866983	newly	45.	40.23	{5., 69.}	96.6889	0.506094	Reff	0.371169	0.421308	{0.0579263, 0.750104}	65.2762	ar	928.	1753.37	{1586., 2118.}	88.9407
46	R0	{2.52291, 18.0251}	{2.58136, 5.69269}	{{1.855, 2.91983}, {3., 8.8115}}	{20.9753, 68.418}	inf	131.	54.0205	{4.82729, 128.184}	82.1449	0.765751	newly	46.	62.16	{3., 165.}	124.435	0.511533	Reff	0.431418	0.493614	{0.149148, 0.833788}	60.7647	ar	894.	1729.49	{1552., 2106.}	93.4553
47	R0	{2.22618, 17.1225}	{2.77895, 4.46097}	{{2.38605, 1.20776}, {3., 6.13571}}	{24.8303, 74.0822}	inf	148.	67.4653	{2.97995, 89.7806}	101.645	0.964047	newly	59.	113.85	{3., 259.}	206.864	0.690717	Reff	0.634461	0.603515	{0.209029, 1.1176}	54.201	ar	850.	1810.87	{1599., 2317.}	113.044
48	R0	{2.04405, 16.073}	{2.78893, 4.26875}	{{2.41102, 1.95505}, {3., 9.0722}}	{36.8614, 73.4415}	inf	103.	39.9632	{3.10682, 44.9351}	88.2468	0.853923	newly	40.	67.12	{3., 118.}	163.2	0.548994	Reff	0.711329	0.548712	{0.210322, 0.902019}	58.8864	ar	779.	1714.69	{1564., 2092.}	120.114
49	R0	{2.84396, 18.097}	{2.5938, 5.11976}	{{1.84993, 1.38068}, {3., 9.238}}	{15.4898, 71.7093}	inf	132.	48.6562	{5.29478, 54.8332}	85.3508	0.779025	newly	43.	47.23	{6., 60.}	85.2326	0.418419	Reff	0.264488	0.441206	{0.0800731, 0.65325}	90.4488	ar	952.	1767.23	{1626., 2043.}	85.6334
50	R0	{1.85341, 18.0975}	{2.77157, 7.79277}	{{2.44989, 2.87333}, {3., 18.5063}}	{50.4358, 57.918}	inf	90.	68.5179	{13.8832, 81.5321}	73.4953	0.404399	newly	26.	56.29	{8., 102.}	154.654	0.42184	Reff	0.615332	0.506827	{0.117439, 0.826351}	56.6413	ar	754.	1783.76	{1613., 1845.}	136.573
